# Clinical effectiveness of implant support for distal extension removable partial dentures: functional evaluation using occlusal force measurement and masticatory efficiency

**DOI:** 10.1186/s40729-021-00381-4

**Published:** 2021-10-11

**Authors:** Kei Murakami, Yasunori Ayukawa, Yoichiro Ogino, Akinari Nakagawa, Tadashi Horikawa, Eishi Yamaguchi, Kimiyasu Takaki, Kiyoshi Koyano

**Affiliations:** 1 Kei Dental Clinic, 1-9-25 Jyozan-ohdomo, Nishi-ku, Kumamoto, 8600067 Japan; 2 Section of Implant and Rehabilitative Dentistry, Division of Oral Rehabilitation, Faculty of Dental Science, Kyushu University, 3-1-1 Maidashi, Higashi-ku, Fukuoka, 8128582 Japan; 3 Section of Fixed Prosthodontics, Division of Oral Rehabilitation, Faculty of Dental Science, Kyushu University, 3-1-1 Maidashi, Higashi-ku, Fukuoka, 8128582 Japan; 4 Nakagawa Dental Clinic, 2377 Sonezaki, Tosu City, Saga 8410025 Japan; 5 Horikawa Dental Clinic, 1-10-23 Saburo, Higashi-ku, Kumamoto, 8620922 Japan; 6 Let’s Dental Clinic, 2-2-18 Shinhoka, Higashi-ku, Kumamoto, 8620921 Japan; 7 Takaki Dental Clinic, 1329-1 Waifu, Kikuchi City, Kumamoto 8611331 Japan; 8 Division of Advanced Dental Devices and Therapeutics, Faculty of Dental Science, Kyushu University, 3-1-1 Maidashi, Higashi-ku, Fukuoka, 8128582 Japan

**Keywords:** Implant-supported removable partial denture, Occlusal force, masticating efficiency

## Abstract

**Background:**

Implant-supported removable partial dentures (ISRPD) are supported at the free-end region with implant retainers. As implant retainers prevent denture settlement and facilitate denture retention, this is intended to improve masticatory performance in comparison with that of conventional removable dentures. In the present study, we evaluated the effect of implant retainers at the free-end region of removable dentures on occlusal force and masticatory efficiency using a pressure-sensitive sheet, and measured glucose concentration in saliva after mastication with gummy candy.

**Methods:**

In the present study, the occlusal force and masticatory efficiency of 13 subjects were measured in the following three conditions: without dentures (Condition 1), wearing dentures but not supported by implants (Condition 2), and wearing dentures supported by implants (ISRPD) (Condition 3). All data were statistically compared.

**Results:**

Regarding the occlusal force, Condition 3 showed significantly higher scores than the other conditions; however, there were no significant differences between Conditions 1 and 2. Regarding the masticatory efficiency, Condition 3 showed significantly higher scores than Condition 2.

**Conclusions:**

With ISRPD, the occlusal force and masticatory efficiency were increased in comparison with those with conventional removable dentures.

## Background

Patients with distal-extension removable partial dentures sometimes complain of discomfort, weak occlusal force, or poor masticatory efficiency [1–3]. When wearing a distal-extension removable denture, abutment teeth are subject to damage accompanied by periodontal bone resorption [4, 5]. This can lead to the loss of a vertical occlusal stop or combination syndrome, creating difficult conditions for dentures. According to a previous report, 25% and 50% of conventional removable partial dentures are no longer in service 5 and 10 years after delivery, respectively [6].

Conversely, patients with fixed implant prostheses were reported to be more satisfied with their comfort, occlusal force, and masticatory efficiency [4, 7, 8]. However, fixed implant prostheses are comparatively expensive, and the condition of severe alveolar ridge resorption requires additional bone-augmentation surgery, necessitating a more invasive and longer treatment period [9, 10]. When patients are of advanced age or have systemic problems or implants that must be removed due to peri-implantitis or overload, a change in the superstructure is sometimes needed.

On the above basis, implant-supported removable partial dentures (ISRPD) were introduced. This prosthetic device is a removable partial denture resting on implants in the distal region, which renders the denture tooth-implant-supported instead of tooth-mucosa-supported; thus, posterior implants generate greater retention, stability, and comfort [11]. In comparison with conventional removable dentures, a case report indicated that ISRPD has the advantages of increased retention, stability, and patient satisfaction [12]. In the case of Kennedy Class I situations, two implants are typically used. In comparison with fixed prostheses, ISRPD can reduce the number of used implants and a superstructure can easily be fabricated; thus, they can reduce costs.

Although various reports indicated success in cases where removable partial dentures were used with implants [13–17], the clinical effectiveness of ISRPD, such as masticatory efficacy and occlusal force, was also reported [18, 19]. Okubo et al. reported in their in vitro study that implant support helped prevent distal-extension removable partial dentures and decreased pressure on soft tissue [20]. In an in vivo study from the same group, ISRPD had greater occlusal force, and the center of occlusal force was distally positioned in comparison with conventional partial dentures. There was also improved patient satisfaction (comfort, chewing, retention, and stability) [21].

In the present study, we investigated the clinical effectiveness of ISRPD regarding occlusal force and masticating efficiency. To measure occlusal force, we employed pressure-sensitive sheets [22]. For evaluating masticatory efficiency, we extracted glucose concentration in the saliva from gummy candy after mastication had been measured [23].

## Materials and methods

Thirteen patients participated in the present study. All participants had free-end missing dentition in either the upper or lower jaw and had removable dentures. Nine patients bilaterally received implants, and four patients unilaterally received implants. As implant attachments, LOCATOR^®^ (Zest dental solutions, Carlsbad, CA, USA; 11 patients) or magnet attachments (Magfit^®^, Aichi Steel, Tokai, Japan; two patients) were used (Table 1). All patients who satisfied these conditions and wanted to have ISRPD treatment were enrolled in the present study. Participants were enrolled from April to September 2020. After at least 2 months of unloaded periods, all implants were confirmed to have secure stability using Periotest^®^ (Medizintechnik Gulden, Modautal, Germany), with a Periotest value of 0 or less.

**Table 1:** Summary of subjects

Case	Age, Sex	Implant position	Manufacturer	Implant	Diameter (mm)	Length (mm)	Attachment	Antagonist dentition
1	64, M	36	Straumann	Tissue level	4.1	8	Locator	Complete denture supported by implants and teeth
		46	Straumann	Tissue level	4.1	8		
2	75, F	35	Zimmer Biomet	Spline Twist	3.75	10	Locator	Natural teeth
		44	Zimmer Biomet	Spline Twist	3.75	10		
3	70, M	36	Zimmer Biomet	Spline twist	3.75	10	Locator	Natural teeth and removable partial denture
		46	Zimmer Biomet	Spline twist	3.75	8		
4	72, M	36	Straumann	Bone level tapered	4.1	10	Locator	Natural teeth
		46	Zimmer Biomet	Spline twist	3.75	10		
5	73, F	36	Straumann	Tissue level	4.1	10	Locator	Natural teeth and removable partial denture
		46	Straumann	Tissue level	4.1	10		
6	61, F	36	Straumann	Tissue level	4.1	6	Locator	Complete denture supported by implants
		46	Straumann	Tissue level	4.1	6		
7	65, F	36	Straumann	Bone level	4.1	8	Magnet	Natural teeth and implant (fixed prosthesis)
		46	Straumann	Bone level	4.8	8		
8	65, F	36	Zimmer Biomet	Spline twist	3.75	10	Locator	Natural teeth and implant (fixed prosthesis)
		46	Zimmer Biomet	Spline twist	3.75	10		
9	69, F	15	Zimmer Biomet	Spline twist	3.75	10	Locator	Natural teeth and removable partial denture
		24	Zimmer Biomet	Spline twist	3.75	10		
10	81, F	16	Zimmer Biomet	Spline twist	3.75	10	Locator	Natural teeth and removable partial denture
11	79, F	25	Straumann	Tissue level	4.1	10	Locator	Natural teeth and implant (fixed prosthesis)
12	60, F	46	Zimmer Biomet	Spline twist	3.75	10	Magnet	Natural teeth and implant (fixed prosthesis)
13	69, F	36	Zimmer Biomet	Spline twist	3.75	10	Locator	Natural teeth

The research protocol was approved by the ethics committee of the Japanese Society of Oral Implantology (Approval number: 2020-1) and complied with the Helsinki Declaration 1964, as revised in 2013.

### Occlusal force

**Fig. 1:**
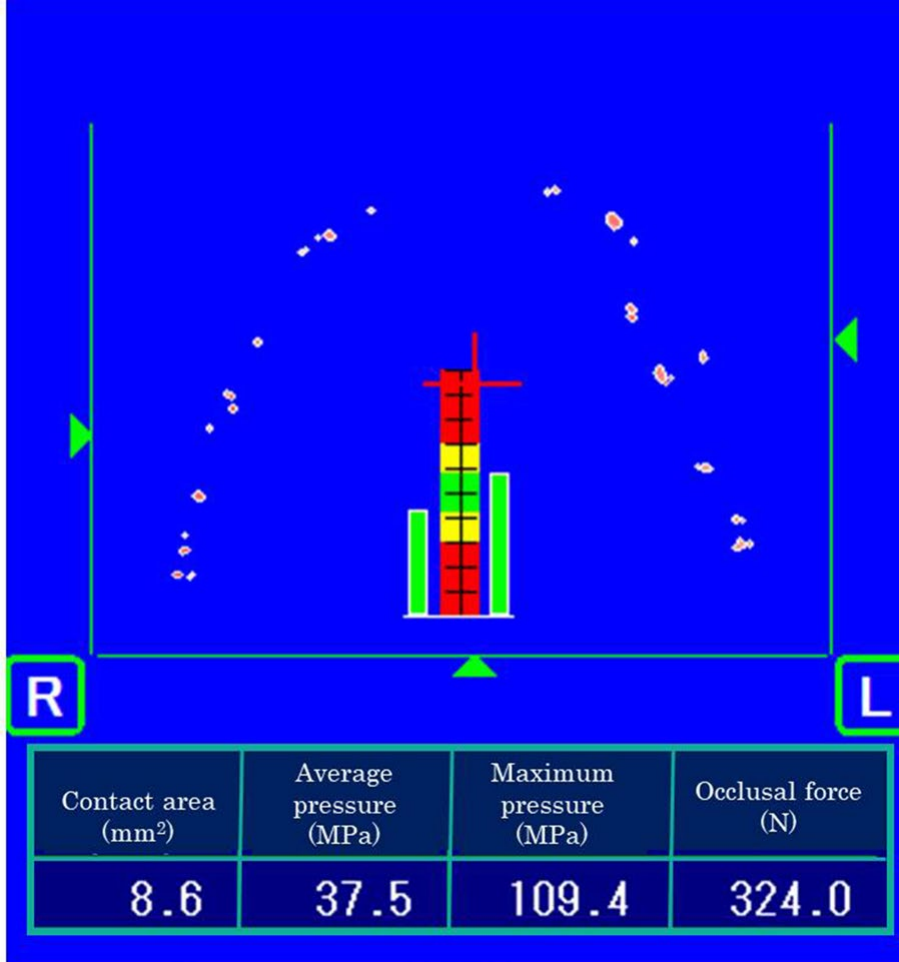
Representative results of analysis of occlusal force

**Fig. 2:**
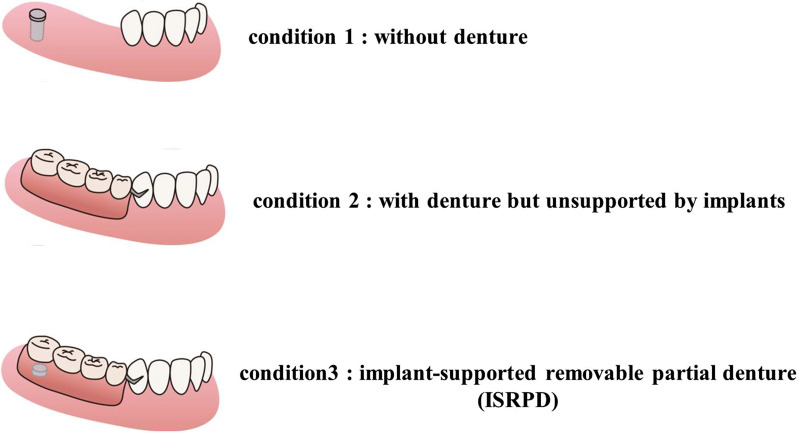
Test conditions

A pressure-sensitive sheet, Dental Prescale^®^ (GC, Tokyo, Japan), was used to measure the occlusal force [22]. This sheet has embedded microcapsules that are broken by pressure. By biting this sheet, contact points between maxillary and mandibular teeth break the embedded microcapsules, releasing the contained dye. These contact points are, therefore, indicated in shades of red (dark red represents strong contact). The visualized contact points on the sheet were scanned using an image scanner (Occluzer FPD 703, Fujifilm, Tokyo, Japan), by which the total occlusal force and occlusal force at the denture or natural tooth areas was measured (Fig. 1). Dental Prescale^®^ sheets were chewed for 3 s in the three following conditions: Condition 1: dentures were not worn; Condition 2: dentures were worn but unsupported by implants (attachments were not installed, and implant and denture base had no contact at the time of measurement); and Condition 3: dentures were supported by implants through attachments (Fig. 2).

In each condition, participants chewed the Dental Prescale^®^ sheets three times. Total occlusal force and the occlusal forces in the denture and tooth regions were measured, and the average values of three sheets in each condition were compared.

### Masticating efficiency

To measure the masticating efficiency, gummy candy (Glucolumn, GC) was masticated for 20 s, followed by a rinse with 10 cc of water for a couple of seconds. The rinse was expelled, collected, and evaluated using a glucose-measuring device (Glucosensor, GC) to measure the amount of glucose in the solution [23]. The mastication of gummy candy was performed in Conditions 2 and 3 as mentioned above. In each condition, the process was repeated twice, and the average values of each test were compared.

### Statistical analyses

An a priori Shapiro–Wilk test was performed to check for normality, and normality was not rejected in all cases (*p* > 0.05). For multiple comparisons, one-way analysis of variance (ANOVA) and a post hoc Tukey test for pairwise comparisons were employed. A paired *t*-test was used for the comparison of two data sets. A value of *p* < 0.05 was considered to be statistically significant. All statistical procedures were performed using a Microsoft Excel statistical add-on (BellCurve for Excel 2.15, Social Survey Research Information, Tokyo, Japan).

## Results

### Occlusal force

#### Total occlusal force

**Fig. 3:**
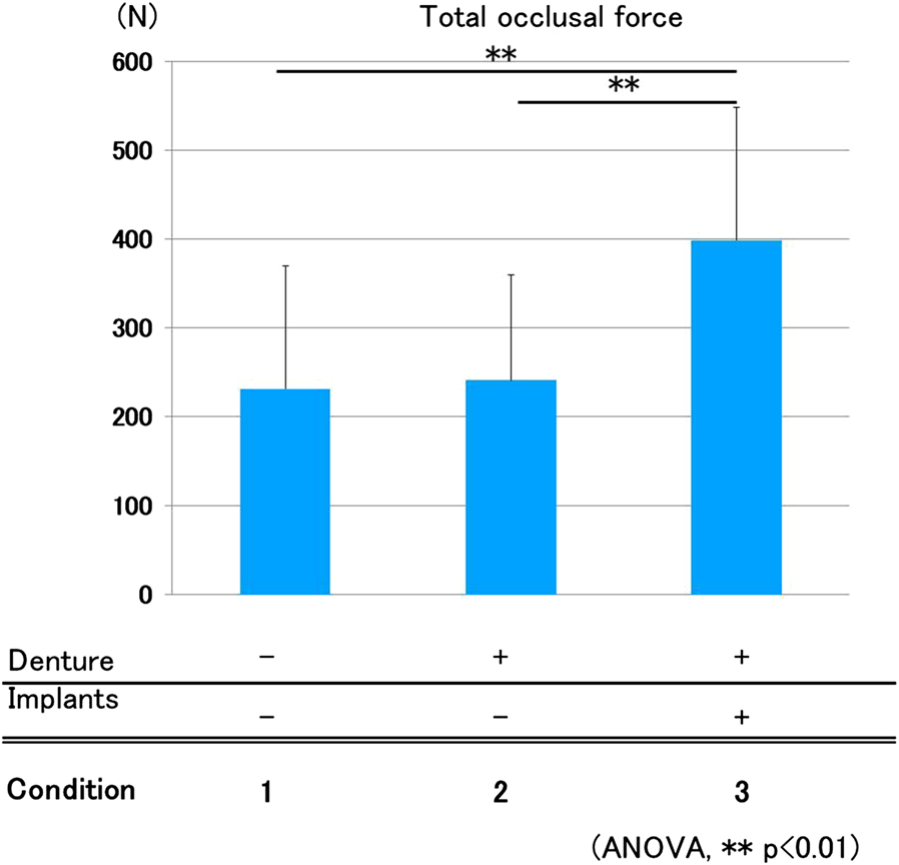
Total occlusal force. Condition 3 (ISRPD) showed significantly greater total occlusal force than that of others (ANOVA, *p* < 0.01)

Average total occlusal force in Conditions 1 (without denture), 2 (with denture without implant retention), and 3 (ISRPD) was 231.1, 240.3, and 398.0 N, respectively. Condition 3 showed significantly higher total occlusal force than that of the others, but there was no significant difference between Conditions 1 and 2 (Fig. 3).

#### Occlusal force at denture area

**Fig. 4:**
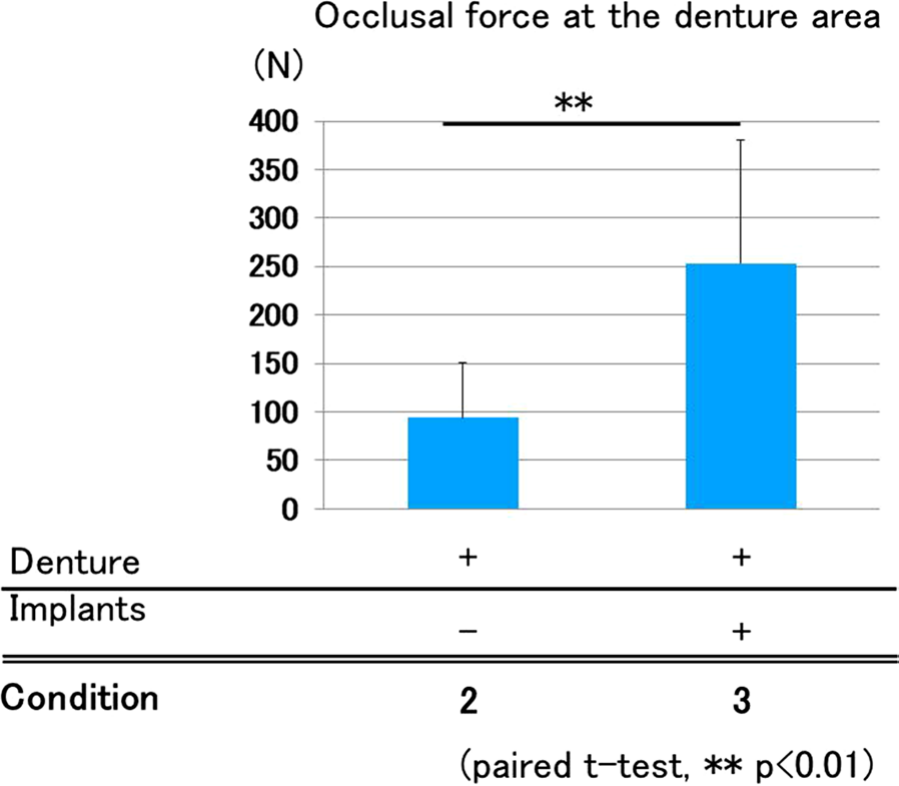
Occlusal force at denture area. Condition 3 (ISRPD) showed significantly greater occlusal force at the denture area compared with Condition 2 (removable partial denture without implant support; paired *t*-test, *p* < 0.01)

The average occlusal force at the denture area in Conditions 2 and 3 was 93.7 and 253.3 N, respectively. ISRPD showed significantly higher occlusal force at the denture area than that of removable partial dentures without implant retention (Fig. 4).

#### Occlusal force at tooth area

**Fig. 5:**
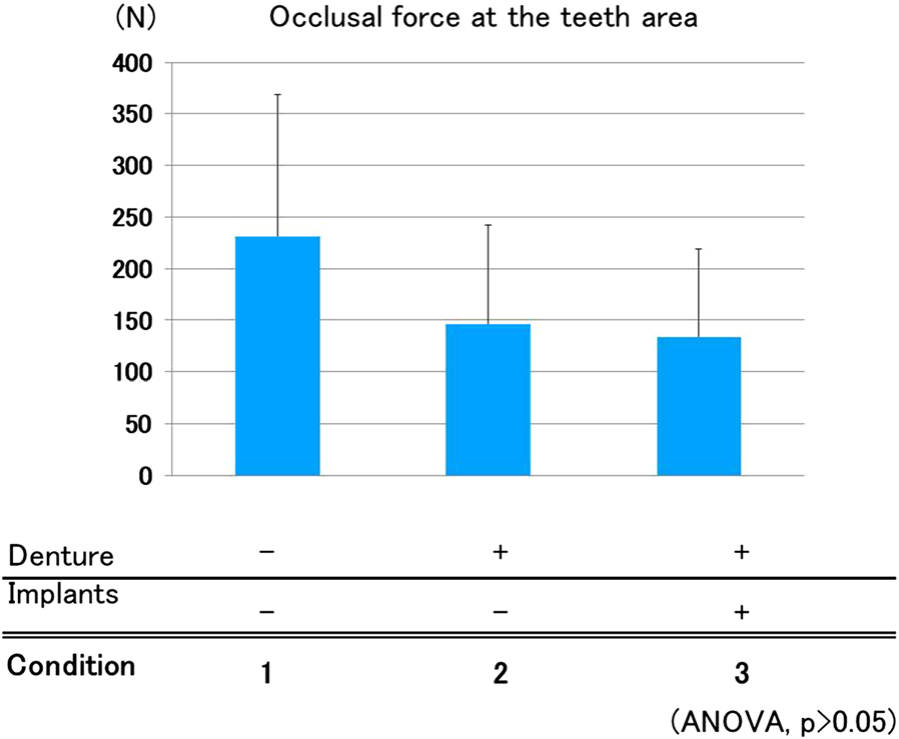
Occlusal force at tooth area. There were no significant differences in occlusal force at the tooth area among the three conditions

Average occlusal force at the tooth area in Conditions 1, 2, and 3 was 231.1, 146.5, and 133.8 N, respectively. Condition 1 showed a higher score than that of the others, but there were no statically significant differences (Fig. 5).

### Masticating efficiency

**Fig. 6:**
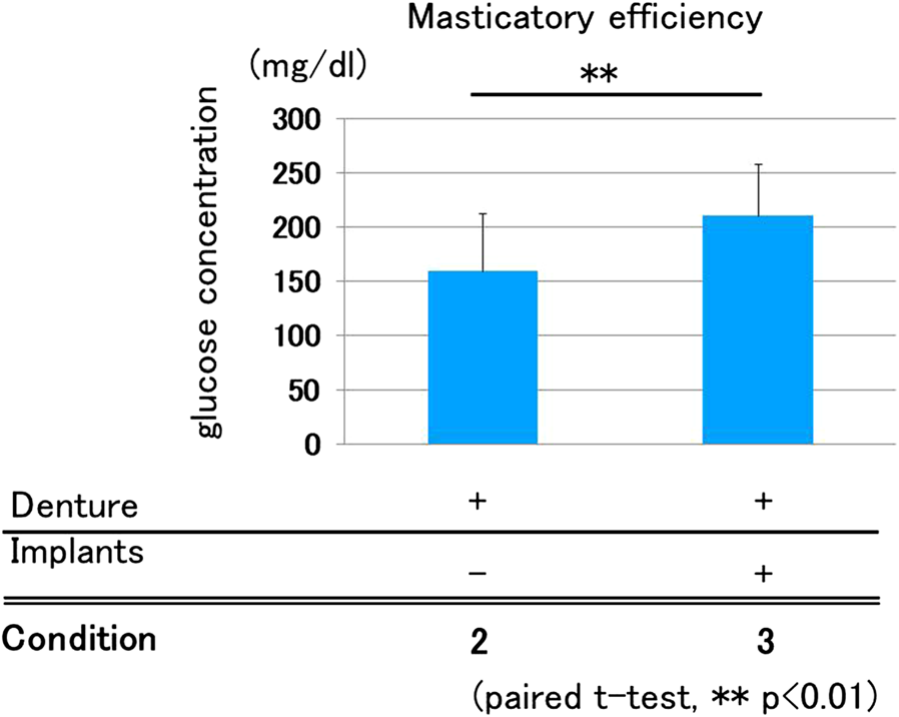
Masticatory efficiency. Condition 3 showed significantly greater masticatory efficiency compared with Condition 2 (paired *t*-test, *p* < 0.01)

Average glucose concentrations in Conditions 2 and 3 were 166.8 and 213.1 mg/dL, respectively. There was a statistically significant difference between them (Fig. 6).

## Discussion

Regarding total occlusal force, in the present study, ISRPD showed significantly higher values than those of the other conditions. This was similar to previously reported data [20, 21]. The support provided from implants may prevent the settlement of the dentures and thereby increase total occlusal force. By contrast, there was no significant difference between dentures without implant support (Condition 2) and the nondenture condition (Condition 1). This implies that total occlusal force was almost the same in these conditions, regardless of the presence or absence of conventional partial dentures. A larger total occlusal force may be the cause of the pain at the denture-bearing mucosa, and may prevent an increase in total occlusal force. Even in this situation, occlusal force was dispersed to the denture area if conventional partial dentures were used (Condition 2). This may prevent the deterioration of periodontal tissue, tooth fracture, or other unexpected events.

Concerning occlusal force in the natural tooth area, although no statistically significant differences were reported, the nondenture condition had a tendency to show a higher score compared with that of the other conditions. According to a previous report, wearing a removable partial denture in free-end partial edentulism may be effective for the preservation of the remaining teeth by reducing excessive stress [24]. This is in accordance with our results indicating that denture wearing reduced the force exerted at the area with the remaining teeth.

Occlusal force exerted at the remaining-teeth area was almost the same between Conditions 2 (dentures) and 3 (ISRPD). In the present study, all dentures were precisely adjusted for occlusion, and this procedure may have equilibrated the force at the remaining-teeth area of both groups. By contrast, force exerted at the denture area was significantly larger in the ISRPD group. This may have been due to the prevention of denture settlement by the presence of implants at the free-end area.

In contrast to our results, in a previous report, there were no significant differences in masticatory performance and occlusal force between implant-supported fixed partial dentures and conventional-removal partial dentures [25]. In the previous study, the lack of significant differences in masticatory performance and occlusal force was speculated to be a consequence of the larger standard deviation of these evaluations. With regard to masticating efficiency, ISRPD showed significantly higher scores than those of the denture-only conditions. This may have been due to the increase in occlusal contact area and the prevention of denture settlement.

Masticatory efficiency was not measured without dentures (Condition 1). In the present study, the concentration of glucose exuded from gummy candy as a consequence of grinding at the occlusion was measured and defined as masticatory efficiency. In the present study, occlusion at the molar regions varied among subjects in association with the number and distribution of the remaining teeth, and this may have been a cause for variation; thus, we did not measure masticatory efficiency without dentures.

In the present study, a small cohort of subjects were retrospectively studied. This is the main limitation of the present study. As ISRPD is still not a first-line implant therapy for partially edentulous dentition, it was difficult to gather a sufficient number of subjects for this study. Thus, this study should be defined as preliminary, and its results should be interpreted with caution. Further prospective research with a larger sample size is expected to elucidate the effect of ISRPD on occlusal performance.

## Conclusion

Within the limitations of the present study, supporting free-end missing removable partial dentures with implants increased both the occlusal force and masticatory efficiency.

## Data Availability

The primary data will be provided from corresponding author upon reasonable request.
